# Resting metabolic rate and its adjustments as predictors of risk protein-energy wasting in hemodialysis patients

**DOI:** 10.1042/BSR20210010

**Published:** 2021-04-30

**Authors:** Jingjing Da, Yanjun Long, Qian Li, Xia Yang, Jing Yuan, Yan Zha

**Affiliations:** 1 School of Medicine, Guizhou University, Guiyang, China; 2 Renal Division, Department of Medicine, Guizhou Provincial People’s Hospital; 3 NHC Key Laboratory of Pulmonary Immunological Disease, Guizhou Provincial People’s Hospital, Guiyang, China

**Keywords:** protein energy wasting, resting metabolism rate, bioelectrical impedance, maintenance hemodialysis

## Abstract

**Background:** The purpose of the present study was to explore the association between resting metabolic rate (RMR) and protein-energy wasting (PEW) risk in Chinese hemodialysis patients by age and gender subgroup.

**Methods:** RMR and body composition (body cell mass (BCM) and fat mass) of 774 patients undergoing hemodialysis were estimated by bio-electrical impedance analysis (BIA). Anthropometric data were collected by a standard measurement protocol, and the upper arm muscle circumference (AMC) was calculated. Biochemical nutritional and dialysis parameters were obtained. Linear regression analysis was used to analyze the relationship among RMR, body composition and nutritional factors.

**Results:** The mean age was 54.96 ± 15.78 years. RMR level in patients was 1463.0 (1240.5, 1669.0) kcal/d. In multiple linear regression models, BCM, left calf circumference (LCC), fat mass were the determinants association with RMR (*P*<0.001). Among the patients in the sample, 133 (17.2%) had been diagnosed with PEW per International Society of Renal Nutrition and Metabolism (ISRNM) criteria and 363 (46.9%) were being at risk PEW. The area under the receiver-operating characteristic curve (AUC) of RMR for predicting risk PEW was greater than RMR/BCM and RMR/body surface area (BSA). When the cutoff of RMR was 1481 kcal/d it had the higher sensitivity and specificity (82 and 42%), and the AUC was 0.68 in elderly maintenance hemodialysis (MHD) patients (*P*<0.001). After adjustment for potential confounders, lowest RMR quartile level (<1239) increased the risk of PEW (OR = 4.71, 95% CI: 1.33–16.64, *P*=0.016) in all patients.

**Conclusions:** Older patients with PEW have a lower RMR reduction. RMR and RMR/BCM may play the role in objective screening to detect risk PEW in MHD patients, especially in males.

## Introduction

The kidney is not only an excretory organ, but also an important energy metabolism organ [1]. Malnutrition and metabolic disorders, referred to as protein-energy wasting (PEW) are common clinical complications in patients with end-stage kidney disease (ESRD) [2,3]. Approximately 18–70% of maintenance hemodialysis (MHD) patients worldwide are suffering from PEW [4], and these data are up to 70–75% in China [5,6]. The main reason of MHD patients with PEW is energy imbalance resulted from malnutrition, inflammation and metabolic acidosis [7,8]. Until recently, concerns are growing about the possibility that alterations in energy consumption may affect energy balance [9]. It was obvious that energy expenditure has the potential relationship with over-nourishment disease, such as diabetes and obesity [10]. Unfortunately, large-scale studies investigating the impact of energy metabolism on malnutrition in dialysis patients have not yet been conducted.

Besides physical activity, basal energy expenditure (BEE) accounts for 60–75% of total daily energy expenditure, and basal metabolic rate (BMR) is a classical index for daily energy expenditure. Currently, resting metabolic rate (RMR) is used to evaluate energy expenditure in clinical practice instead of BMR due to being easily detected and only 10% higher [11]. Bio-electrical impedance analysis (BIA) is a rapid, reliable and noninvasive method that has been used for assessment of BMR in type 2 diabetes, children with cerebral palsy and sarcopenia in older males [12–14]. Notwithstanding, age-related changes in body composition may have negative effects on the functional status of the elderly, including a gradual decline in muscle mass, strength and mass, as well as an increase in fat mass [15,16]. Therefore, effective management strategies of energy expenditure for elderly dialysis patients with PEW need to be fully understood. Given the evidence that RMR decreased with aging or weight loss, we hypothesized that the association of RMR on nutritional status and anthropometry could differ by age group or gender group in dialysis patients. Therefore, the present study compared the level of RMR based on bio-electrical impedance in 11 hemodialysis centers in Guizhou Province, and demonstrated the ability of RMR to predict PEW in MHD patients between age and gender subgroup analysis.

## Materials and methods

### Participants

Patients aged 18–90 years underwent routine MHD at 11 dialysis centers in Guizhou, Southwest China, were enrolled in this multicenter, prospective study. Inclusion criteria: (i) dialysis facilities chosen from Grade III general hospitals and voluntary participation in the study, (ii) patients were assessed by BIA and regular hemodialysis three times a week for 4 h each time. We excluded patients who did not sign the informed consent, had acute illness in the last 3 months, severe sepsis, shock, multiple organ dysfunction syndrome (MODS) and recent change in dialysis modality.

All patients were dialyzed using 1.6 m^2^ surface area high-flux polysulfone dialyzers with bicarbonate-based dialysate (Na^+^ 138 mmol/l, HCO^3–^ 32 mmol/l, K^+^ 2.0 mmol/l, Ca^2+^ 1.50 mmol/l, Mg^2+^ 0.5 mmol/l). The prescribed duration time was 4 h with a blood flow rate of 250 ml/min and a dialysate flow rate of 500 ml/min. The adequacy of dialysis was assessed by using *K*_t_/V (K, dialyzer clearance of urea; t, dialysis time; V, volume of distribution of urea). According to Kidney Disease Outcomes Quality Initiative (KDOQI) 2015 update, the target *K*_t_/V should be 1.2 per session.

### Anthropometric measurements

To evaluate nourishment status in the subjects, we assessed their height (cm) and weight (kg) with light clothes. Body mass index (BMI) was calculated as weight/height^2^ (kg/m^2^). Upper arm circumference (AC) and left calf circumference (LCC) was measured with soft feet. The measuring point of AC was the midpoint from shoulder to olecranon connection with upper limbs naturally drooping, and that of LCC was the strongest level of left calf. Triceps skinfold thickness (TSF) was performed with calliper and read the needle to the nearest 0.1 mm approximately. To minimize intra-operator variability, the averages of three consecutive measurements were recorded. Upper arm muscle circumference (AMC) was calculated as AC − 3.14 × TSF. Grip strength (GS) was obtained using a hand-held dynamometer. The patient squeezed the dynamometer with all of their strength, typically three times with left hand. Body surface area (BSA, m^2^) was calculated as 0.0061 × height + 0.128 × weight − 0.1529.

### Body composition measured and predictive equations of RMR

Body capacitance, lean body mass (LBM), fat mass, body cell mass (BCM), intracellular water (ICW) and RMR were assessed using a five-compartment model of bio-electrical impedance analyzer (Biodynamic BIA 450, WA, U.S.A.) with an electrical current of 50 kHz. Measurement was performed before a mid-week dialysis session on the non-access site of the body. Patients were laid in the quiet room with the temperature 22–24°C. Two pairs of sensor electrodes were placed on the patient’s right hand and wrist, and right foot and ankle.

### Laboratory parameters

Blood samples were also obtained in the morning after an 8-h overnight fast. Laboratory parameters included: pre-dialysis serum creatinine (Scr), blood urea nitrogen (BUN), parathyroid hormone (PTH). Biochemical indicators were tested by automatic biochemical detector (Olympus, Tokyo, Japan).

### Definition of risk PEW

Diagnostic criteria provided by the International Society of Renal Nutrition and Metabolism (ISRNM) were used to identify risk PEW in our patients. In the present study, serum albumin <38 g/l, BMI < 23 kg/m^2^, AMC < 19.62 cm (reduction > 10% in relation to 50^th^ percentile of overall hemodialysis patients) were classified for the presence of risk PEW. According to the number of items that meet the diagnostic criteria, we defined the mild risk of PEW (one out of three categories), moderate risk of PEW (two categories) and diagnosed PEW (more than or equal to three categories) [17].

### Statistical analysis

SPSS software (IBM, Chicago, IL) were used for statistical analyses. Means ± SDs or median with interquartile range were used for normally and non-normally descriptive statistics of continuous variables, respectively. Two-way Multiple Analysis of Variance (two-way MANOVA) was used to find the difference of RMR in the groups cross-classified by age and gender. Skewed data among the groups were analyzed using Kruskal–Wallis test*.* Categorical variables were estimated for association of RMR and PEW by the chi-square test. In multivariate linear regression analysis, RMR was modeled as the independent variable and other variables with *P*≤0.10 were inserted. The receiver-operating characteristic (ROC) curve was carried out for the assessment of accuracy of diagnostic tests. Associations of RMR with presence of PEW risk was performed by logical regression. Throughout the study, *P*<0.05 was taken as the minimum level of statistical significance.

## Results

### Profile of the patients

**Figure 1:**
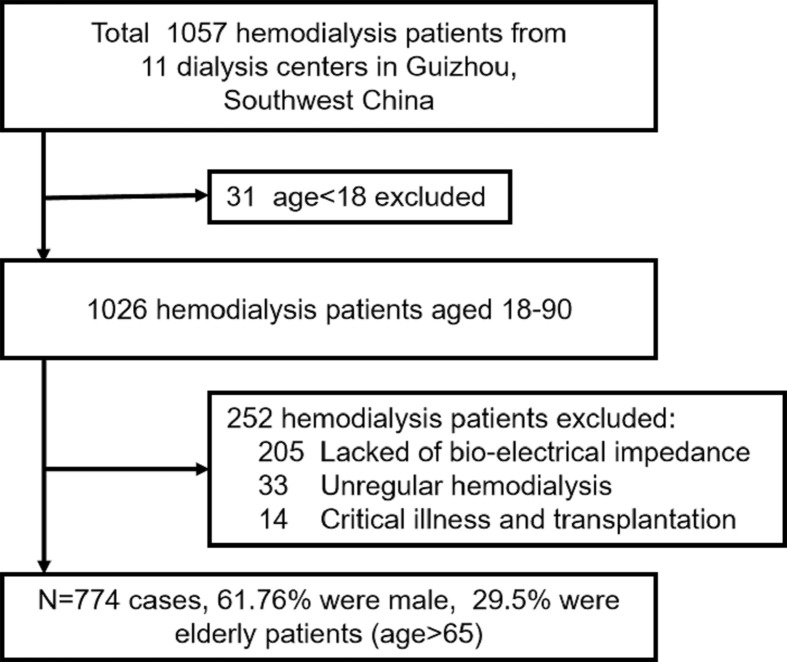
A flow chart of patients enrolled

Figure 1 showed that a total of 774 patients underwent hemodialysis with a mean of 54.96 ± 15.78 years (range: 18–90 years) and a mean duration of 42.33 ± 9.0 months were enrolled from 11 dialysis centers of Guizhou in southwest China. Of all patients, 228 cases (29.5%) were elderly patients. The primary causes of chronic kidney disease (CKD) included chronic glomerulonephritis (402 cases, 51.94%), diabetic kidney disease (143 cases, 18.48%), hypertension nephropathy (137 cases, 17.70%), congenital obstructive nephropathy (18 cases, 2.33%), polycystic kidney (8 cases, 1.03%), others (66 cases, 8.53%).

### The incidence of risk PEW in hemodialysis patients

Only 133 cases (17.2%) were diagnosed with PEW per ISRNM criteria; 33.1% of cases were classified as having moderate risk of PEW and 13.8% of cases were classified as being at mild risk of PEW (met one of four PEW criteria) and 35.9% of subjects fulfilled no PEW criteria (Table 1). Compared with younger patients, older patients more often had disorder in these criteria (X^2^ = 8.228, *P*=0.042), and there was no statistical significance between gender groups (X^2^ = 3.199, *P*=0.362).

**Table 1:** The incidence of risk PEW in hemodialysis patients

	All (*n*=774)	Age		Gender	
		Age < 65 (*n*=546)	Age ≥ 65 (*n*=228)	Male (*n*=478)	Female (*n*=296)
No PEW, *n* (%)	278 (35.9)	208 (38.1)	70 (30.7)	176 (36.8)	102 (34.5)
Mild risk, *n* (%)	107 (13.8)	79 (14.5)	28 (12.3)	72 (15.1)	35 (11.8)
Moderate risk, *n* (%)	256 (33.1)	164 (30.0)	92 (40.4)	154 (32.2)	102 (34.5)
PEW, *n* (%)	133 (17.2)	95 (17.4)	38 (16.7)	76 (15.9)	57 (19.3)
		X^2^ = 8.228, *P*=0.042		X^2^ = 3.199, *P*=0.362	

### Anthropometric data, body compositive characteristics of patients categorized by age and gender

Data are shown in Table 2. RMR level in younger group (age < 65) was 1641.0 (1488.0, 1795.0) kcal/d for male and 1240.5 (1120.0, 1392.0) kcal/d for female, and in elderly group, 1498.0 (1358.5, 1671.5) kcal/d for male and 1192.0 (1064.0, 1304.8) kcal/d for female. The latter in elderly group was the lowest level of RMR. There was a statistically significant interaction between age and gender groups (*P*<0.05). However, statistical differences in RMR levels between age and gender were eliminated after adjustment for LBM. Compared with the younger group, elderly patients had a lower serum creatinine, body capacitance, BCM, LBM, ICW, LCC and GS, there were statistically significant interaction between age and gender groups (*P*<0.05). On the other hand, female in elderly group had a greater fat mass than men and both in younger group (*P*<0.05). In additional, age and BMI were higher, and PTH was lower in elderly group compared with younger group, but there were no statistical significance between gender groups. Dialysis duration, *K*_t_/V, BUN, AMC and TSF no statistically significant interaction between age and gender groups (*P*>0.05).

**Table 2:** Anthropometric data, body composition of hemodialysis patients, grouped according to age and gender

	All (*n*=774)	Age < 65		Age ≥ 65		*P*-value^1^
		Male (*n*=330)	Female (*n*=216)	Male (*n*=148)	Female (*n*=80)	
Age, y	54.96 ± 15.78	47.03 ± 11.76	45.98 ± 11.97	72.47 ± 5.43	72.52 ± 5.45	A
Dialysis Duration, m	42.33 ± 9.00	43.16 ± 9.02	42.27 ± 9.56	43.45 ± 9.23	42.76 ± 9.45	NS
*K*_t_/V	1.38 ± 0.28	1.39 ± 0.48	1.38 ± 0.55	1.37 ± 0.49	1.39 ± 0.61	NS
Scr, μmol/l	889 (646, 1128)	1108 (875, 13550)	864 (703, 1069)	799 (5920, 1047)	706 (431, 896)	A×G
BUN, mmol/l	21.18 (15.69, 25.99)	23.92 (18.00, 28.46)	22.06 (17.76, 26.17)	20.03 (14.90, 25.54)	16.53 (11.02, 22.73)	NS
Capacitance, F	609 ± 219	668 ± 252	588 ± 175	604 ± 217	551 ± 162	A×G
BCM, kg	22.18 ± 6.65	26.22 ± 5.88	19.24 ± 4.86	22.96 ± 6.79	17.04 ± 3.56	A×G
LBM, kg	46.80 (39.60, 53.45)	52.40 (47.50, 57.65)	39.75 (35.93, 44.80)	47.95 (43.55, 53.35)	38.10 (33.88, 41.58)	A×G
Fat mass, kg	12.60(8.00, 17.35)	9.55(5.63, 14,55)	13.30 (9.43, 16.80)	14.20 (10.70, 18.33)	16.60 (11.63, 22.38)	A×G
BMI, kg/m^2^	23.00 (20.75, 25.30)	23.00 (20.70, 25.00)	22.20 (20.03,24.70)	23.40 (21.10, 25.40)	23.90 (21.15, 26.68)	A
RMR, kcal/d	1463.0 (1240.5, 1669.0)	1641.0 (1488.0, 1795.0)	1240.5 (1120.0, 1392.0)	1498.0 (1358.5, 1671.5)	1192.0 (1064.0, 1304.8)	A×G
RMR/LBM, kcal/kg	31.200 (31.195, 31.205)	31.200 (31.196, 31.205)	31.200 (31.195, 31.207)	31.200 (31.194, 31.205)	31.200 (31.192, 31.205)	NS
ICW, kg	19.38 ± 5.33	23.09 ± 4.63	16.77 ± 3.86	20.11 ± 4.66	14.63 ± 2.71	A×G
AMC, cm	25.50 (23.50, 27.50)	22.67 (21.10, 24.30)	20.33 (18.82, 22.57)	22.23 (20.96, 23.86)	20.80 (19.17, 21.98)	NS
LCC, cm	32.5 (30.5, 34.5)	33.5 (31.5, 35.5)	32.0 (29.5, 34.0)	32.0 (30.2, 34.0)	30.5 (28.8, 33.0)	A×G
TSF, mm	11.0 (7.0, 15.0)	9.0 (6.0, 12.0)	13.5 (10.0, 18.5)	9.0 (6.5, 12.0)	12.8 (11.0, 19.3)	NS
GS, kg	21.20 (14.60, 28.60)	28.80 (21.35, 34.53)	17.70 (12.80, 21.40)	21.60 (16.50, 26.00)	11.40 (8.25, 14.65)	A×G
PTH	214.80 (63.11, 485.93)	281.20 (66.83, 537.65)	241.90 (50.40, 601.30)	195.10 (79.55, 347.50)	120.25 (33.89, 346.62)	A

1 *Two-way MANOVA*. Significant (*P*<0.05) effects are given for age (A), gender (G), and interaction age with gender (A×G).

### Multivariate linear regression analysis for RMR in MHD patients by age and gender subgroup

To further investigate the independent correlation of RMR in different individuals, multiple linear regression models were constructed using a model adjusted for LBM, BMI, PTH, GS and ICW. It revealed that BCM, LCC, capacitance and fat mass (Standardized *β* = 0.646, 0.198, 0.120 and −0.122, respectively, *P<0.001*) values maintained independent factors association with RMR in all patients. Same factors were also the independent determinants of RMR in male, age < 65, as well as age ≥ 65 patients, data are shown in Supplementary Table S1. However, in female patients, besides BCM, LCC and fat mass, serum creatinine (Standardized *β* = −0.103, *P<0.05*) instead of capacitance was the different determinant association with RMR (Supplementary Table S1).

### Resting metabolism rate as a predictor of PEW in hemodialysis patients

**Figure 2:**
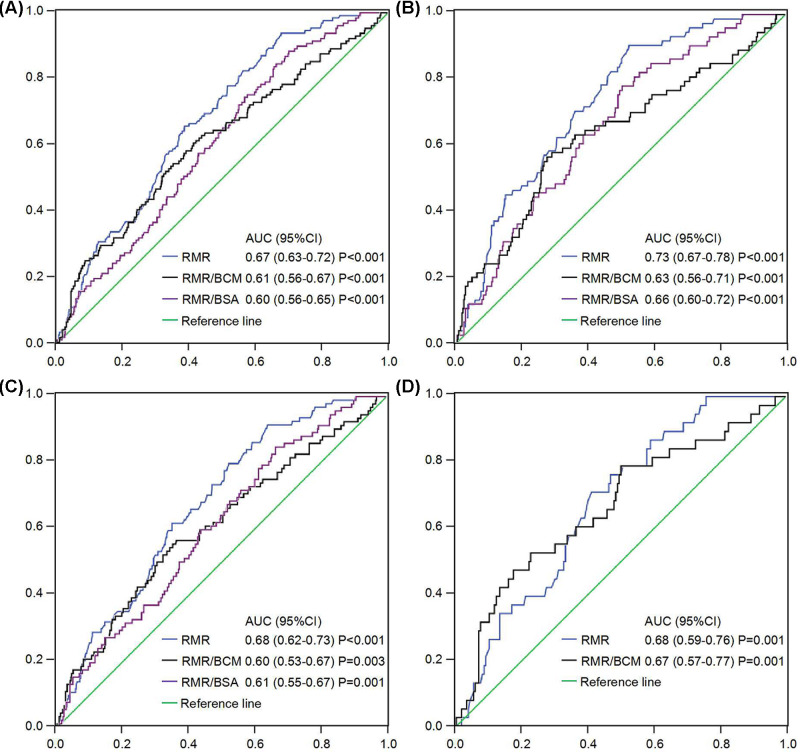
RMR and its adjustment for predicting PEW in MHD Performance of RMR and its adjusted value by BCM or BSA for predicting PEW in all MHD subjects (**A**), male (**B**), age < 65 (**C**) and age ≥ 65 (**D**). For predicting PEW, the AUC of RMR in male MHD patients was the greatest among tested biomarkers, and greater than RMR/BCM and RMR/BSA. However, the AUC of RMR/BSA [0.59 (0.50–0.68)] in elderly patients did not reach statistical significance (*P*=0.081).

For predicting PEW, the area under the receiver-operating characteristic curve (AUC) and cut-off value of RMR for males were 0.73 and 1628 kcal/d (sensitivity 0.85, specificity 0.50), for females 0.65 and 1278 kcal/d (sensitivity 0.77, specificity 0.41), respectively. In age subgroups, 0.68 and 1481 kcal/d (sensitivity 0.82, specificity 0.42) for elderly patients and 0.68 and 1609 kcal/d (sensitivity 0.86, specificity 0.40) for age < 65 patients, respectively (*P*<0.01). As shown in Figure 2, the AUC of RMR for predicting PEW was the greatest among the adjusted RMR, such as RMR/BCM and RMR/BSA in HD patients. The cutoff, sensitivity and specificity of RMR and its adjusted value for predicting PEW were displayed in Supplementary Table S2, we found that RMR remained the higher sensitivity and specificity among the adjusted RMR when the cutoff was 1628 kcal/d in male patients.

After adjusting for potential confounders (age, gender, dialysis duration, fat mass, Scr, BCM, left calf circumference and GS), data in Table 3 demonstrated consistent correlations between RMR and the presence of risk PEW. Lowest RMR level (<25^th^ percentile, 1239) increased the risk of PEW in this group (odds ratio [OR] = 4.71, 95% CI: 1.33–16.64, *P*=0.016), reduced risk with higher RMR level (50–75^th^ percentile, 1463–1669) was also statistically significant (OR = 3.10, 95% CI: 1.14–8.45, *P*=0.027).

**Table 3:** Associations of RMR with presence of PEW in hemodialysis patients

	*n*	*n* (%) PEW	Unadjusted	*P*	Model 1*	*P*	Model 2^∧^	*P*
Quartile 1, <1239 kcal/d	194	49 (25.3)	7.69 (3.53, 16.75)	<0.001	11.59 (4.88, 27.50)	<0.001	4.71 (1.33, 16.64)	0.016
Quartile 2, 1239–1463 kcal/d	202	44 (21.8)	6.34 (2.90, 13.86)	<0.001	7.81 (3.47, 17.60)	<0.001	3.49 (1.15, 10.61)	0.028
Quartile 3, 1463–1669 kcal/d	188	31 (16.5)	4.49 (2.01, 10.06)	<0.001	4.58 (2.04, 10.27)	<0.001	3.10 (1.14, 8.45)	0.027
Quartile 4, >1669 kcal/d	190	8 (4.2)	Reference		Reference		Reference	

Model 1*, adjusted age and sex; Model 2^∧^, additionally adjusted for Scr, BCM, fat mass, GS and left calf circumference.

## Discussion

Energy monitoring for disease state has become an important indicator of guiding the nutritional support treatment [2]. In general, large scale of clinical epidemiological research preferred using predictor formula to direct measurement for energy expenditure assessment [18]. This method mostly uses indirect calorimetry to determine RMR of healthy population, and then establishes predictor formula according to gender, weight, height and age, including Harris–Benedict (H-B) formula, Schofield formula, Adjust-DRI formula, FAO/WHO/UNU formula and the Liu’s formula [19]. However, those RMR predictor formula based on the healthy body can not truly reflect energy expenditure of the patients in disease state [20]. Rigalleau et al. evaluated the REE level by indirect calorimetry in healthy people, diabetes, uremia and diabetic nephropathy, and compared with predicted REE by H-B formula, found that predicted REE in diabetes was underestimated and in uremia was overestimated, and individual differences and poor consistency of REE were found in patients with diabetic nephropathy [21]. In addition, the distribution of water in the body will also be significantly changed during malnutrition, which is often manifested as loss of intracellular fluid, increase of the ratio of extracellular to intracellular fluid.Therefore, comprehensively considering fluid overload in hemodialysis patients [22], it is critical to evaluate the RMR level with bio-electrical impedance analyzer (BIA).

Our previous studies have found that RMR levels in patients with MHD were associated with anthropometry level, such as calf circumference and AMC, which may become a new index to evaluate energy consumption and malnutrition [23,24]. Body composition parameters, such as weight, fat mass and muscle mass (also called LBM), are important indicators for the assessment of energy expenditure [25]. In this study, we focused on the determinants of RMR in elderly dialysis patients between gender and found that RMR is positively related to BCM and LCC, while shows negative association with fat mass both in male and female elderly MHD patients.When shortage of energy in healthy bodies, fat oxidation is the main source of energy supply. However, in dialysis patients with abnormal energy metabolism, it is often manifested as persistent protein degradation and progressive tissue consumption, including proportional reduction in fat and lean mass, as well as BCM [25]. The latter reflects the number of functional cell mass involved in O_2_ consumption, CO_2_ production and energy expenditure in the human body [26]. The main result of Rondanelli et al.’s study was shown that BCM index (BCMI) as a prognostic index of malnutrition and muscle mass status in geriatric population [27]. Another study also shown that BCM was strongly associated with biochemical determinants of muscle mass (Scr) and muscle function (GS) in patients treated with HD [28]. Our study analyzes for the first time to explore lower RMR was strongly associated with BCM in older MHD patients.

Additionally, there is no consensus about whether gender affects RMR, which is especially scare in elderly patients with ESRD [29]. It was found that RMR, LBM and BCM in male dialysis patients are significantly higher than those of females. After adjusting the LBM, the difference of RMR level between genders disappeared, indicating that gender has no influence on RMR in patients with hemodialysis. The research conducted by Buchholz et al. was consistent with the results of the present research [30]. However, there are gender differences in the determinants of RMR in elderly patients in our study. RMR was also significantly associated with serum creatinine in older women and capacitance in older men patients. Creatinine and urea are catabolites of tissue protein and of muscular energetic metabolism [31]. In clinical, serum creatinine is used to evaluate the level of urinary toxins in patients with renal failure, while the latter is the general term for metabolic wastes in metabolic activities. Capacitance is considered to be a physical quantity. In biological systems membranes serve as ‘capacitors for energy and metabolism’ [32], and there was a research displayed that the capacitance of cell membrane as a prognostic indicator of survival in head and neck cancer [33]. In our study, RMR was significantly associated with capacitance in older men patients, indicated that the cell membrane was the primary and critical regulator of resting energy expenditure, which may be explained by decreasing the activity of Na/K-ATPase and the turnover rate of skeletal muscle protein, potential changes of proton permeability in mitochondrial membrane.

A large number of studies have shown that the high prevalence of PEW among HD patients ranged from 31 to 75% when separately assessed with different nutritional methods, including the malnutrition inflammation score and SGA questionnaire [34]. When using the criteria established by ISRNM expert panel, the prevalence in European CONTRAST study was 74% and more tightly decreased at ∼40% in a Spanish study [35,36]. However, in our study only 133 cases (17.2%) were diagnosed with PEW per ISRNM criteria and 35.9% of subjects fulfilled no PEW criteria. A similar result has been found in a Japanese study was 15% of their HD patients met PEW criteria lower than that has been seen in previous [37]. It may be the result of differences between the countries in which these studies were conducted, socioeconomic status, co-morbidity, different lifestyles, sample heterogeneity and medical care in different countries or even in the same national hospital [35]. It is worth noting that, 46.9% of cases were classified as being at mild or moderate risk of PEW (met one or two of four PEW criteria), emphasizing the high nutritional risk of the population studied. With aging, alteration in RMR is usually associated with chronic disease or inflammation status, but it is not known whether RMR can predict PEW in MHD patients. In our study, the AUC of RMR for predicting PEW was the greatest among the adjusted RMR, such as RMR/BCM and RMR/BSA in hemodialysis patients, especially in male patients was 0.73, and RMR remained the highest sensitivity and specificity (0.85 and 0.50) when the cut-off value was 1628 kcal/d.

Some weaknesses and limitations in the present research should be noted. Firstly, indirect calorimetry is recognized as ‘the gold standard’ which can determine energy wasting. The RMR in the present study is obtained based on the analysis of BIA, which eliminates the effect of fluid loading on RMR. However, whether this method is specific to hemodialysis patients remains to be further studied. Finally it is important to point out that a gender difference in the prediction of PEW in RMR assessed by BIA after adjustment of BCM and BSA, and the sensitivity of male is higher than that of female. Therefore, it is necessary to further explore and improve the indicators for predicting PEW in female patients.

## Conclusion

In the present study, we have found that older patients with PEW have a lower RMR reduction. RMR and RMR/BCM may play the role in objective screening to detect PEW in MHD patients, especially in males. The current study is valuable because it is one of the largest scale studies to explore the relationship of RMR and PEW in MHD patients.We have evaluated the cut-off value for screening PEW in MHD, which will be a noteworthy tool in clinical practice.

## Supplementary Material

Supplementary Tables S1-S2

## Data Availability

All the data herein described is publicly available in a Mendeley Data archive ‘Anthropometric and Body composition data of multicenter hemodialysis patients in Guizhou, China’, Mendeley Data, V3, doi: 10.17632/cv4zkd8f35.3.

## Abbreviations

AC: upper arm circumference
AMC: upper arm muscle circumference
AUC: area under the receiver-operating characteristic curve
BCM: body cell mass
BIA: bio-electrical impedance analysis
BMI: body mass index
BMR: basal metabolic rate
BSA: body surface area
BUN: blood urea nitrogen
ESRD: end-stage kidney disease
GS: grip strength
H-B formula: Harris–Benedict formula
ICW: intracellular water
ISRNM: International Society of Renal Nutrition and Metabolism
*K*_t_/V: K, dialyzer clearance of urea; t, dialysis time; V, volume of distribution of urea
LBM: lean body mass
LCC: left calf circumference
MHD: maintenance hemodialysis
PEW: protein-energy wasting
PTH: parathyroid hormone
RMR: resting metabolic rate
Scr: serum creatinine
TSF: triceps skinfold thickness
