# Supplementary material for: Resting metabolic rate and its adjustments as predictors of risk protein-energy wasting in hemodialysis patients
Source: Biosci Rep. 2021 Apr 30;41(4):BSR20210010. doi: 10.1042/BSR20210010 (PMC8150161; doi:10.1042/BSR20210010)
Supplement: Supplementary Tables S1-S2 [file BSR-2021-0010_supp.pdf]

Table S1 Multivariate linear regression analysis using rest metabolic rate in hemodialysis patients

	All (R^2 =0.796, N=774)			Age<65 (R^2 =0.770, N=546)			Age≥65 (R^2 =0.877, N=228)			Male (R^2 =0.745, N=478)			Female (R^2 =0.720, N=296)		
	β	β1	*P*	β	β1	*P*	β	β1	*P*	β	β1	*P*	β	β1	*P*
BCM, kg	30.61 (27.91,33.31)	0.646	<0.001	29.46 (26.14,32.77)	0.635	<0.001	34.21 (29.81,38.42)	0.657	<0.001	24.44 (21.23,27.65)	0.585	<0.001	24.76 (19.09,30.44)	0.448	<0.001
LCC, cm	12.86 (9.17,16.55)	0.198	<0.001	10.97 (6.71,15.24)	0.185	<0.001	26.49 (18.97,34.00)	0.302	<0.001	12.17 (8.24,16.10)	0.239	<0.001	17.86 (10.32,25.41)	0.285	<0.001
Fat mass, kg	-4.69 (-6.66,-2.73)	-0.122	<0.001	-4.24 (-6.75,-1.73)	-0.110	0.001	-6.33 (-9.25,-3.41)	-0.164	<0.001	-5.01 (-7.27,-2.76)	-0.159	<0.001	-	-	-
Capacitance, F	0.17 (0.10,0.25)	0.120	<0.001	0.13 (0.03,0.23)	0.091	0.009	0.22 (0.11,0.33)	0.153	<0.001	0.13 (0.04,0.21)	0.112	0.003	0.20 (0.07,0.34)	0.152	0.003
Scr, umo/L	-	-	-	-	-	-	-	-	-	-	-	-	-0.07 (-0.14,-0.01)	-0.103	0.036

Abbreviation: β 1, standardized coefficient; BCM, body cell mass; LCC, left calf circumference; Scr, serum creatinine.

Table S2 RMR and its adjusted value for predicting PEW risk in hemodialysis patients

	All			Age<65			Age⩾65			Male			Female		
	Cut-off	Sensitivty	Specificity	Cut-off	Sensitivty	Specificity	Cut-off	Sensitivty	Specificity	Cut-off	Sensitivty	Specificity	Cut-off	Sensitivty	Specificity
RMR, kcal/d	1569	0.84	0.40	1609	0.86	0.40	1481	0.82	0.42	1628	0.85	0.50	1278	0.77	0.41
RMR/BCM, kcal/kg	66	0.72	0.41	65	0.71	0.42	69	0.79	0.41	65	0.74	0.41	-	-	-
RMR/BSA, kcal/m^2	960	0.76	0.40	974	0.75	0.40	-	-	-	997	0.84	0.41	-	-	-

Abbreviation: RMR, resting metabolic rate; BCM, body cell mass; BSA, body surface area.
